# Chemical diversity in leaf and stem essential oils of *Origanum vulgare* L. and their effects on microbicidal activities

**DOI:** 10.1186/s13568-019-0893-3

**Published:** 2019-10-31

**Authors:** Merajuddin Khan, Shams T. Khan, Mujeeb Khan, Ahmad A. Mousa, Adeem Mahmood, Hamad Z. Alkhathlan

**Affiliations:** 1 Department of Chemistry, College of Science, King Saud University, P.O. Box 2455, Riyadh, 11451 Saudi Arabia; 2 Department of Agricultural Microbiology, Aligarh Muslim University, Aligarh, 202002 U.P India

**Keywords:** Essential oils, NMR, GC–MS, *Origanum vulgare* L., Lamiaceae

## Abstract

Essential oils (EOs) from the stems and leaves of *Origanum vulgare* L. grown in Saudi Arabia and Jordan were analyzed by gas chromatography–mass spectrometry (GC–MS) and GC–flame ionization detector (FID) techniques on two different columns (polar and nonpolar). A detailed phytochemical analysis led to the identification of 153 constituents of these essential oils. Both Saudi and Jordanian plants are classified by chemotypes rich in cymyl-compounds. However, the Saudi *Origanum* contains carvacrol as the major component and is, thus, characterized as a carvacrol chemotype, while the Jordanian *Origanum* contains thymol as the major component, and, thus, it is classified as a thymol chemotype. In addition, the antimicrobial activities of the studied EOs and their major components, including carvacrol and thymol, were evaluated against various Gram-positive and Gram-negative microorganisms. All the tested compounds exhibited significant antimicrobial activity against all the tested bacteria. Among them, thymol demonstrated superior activity against all the tested organisms, followed by carvacrol. Moreover, results on oil composition and oil yield of *O. vulgare* L. from different parts of the world is compared in detail with the present outcomes.

## Introduction

Recently, the demand for the development of natural products from medicinal and aromatic plants as substitutes for artificial additives and as pharmacologically active agents has increased significantly (Atanasov et al. 2015). Among the different natural products, essential oils (EOs) have gained immense popularity in various industries, including the food, cosmetics, and pharmaceutical industries, because of their remarkable characteristics such as, strong odor, unique colors, and high volatility (Carvalho et al. 2016; Maggio et al. 2016). In particular, EOs play a significant role in the health care sector by virtue of their remarkable biological activities, which are directly associated with their biologically active essential oil components (Raut and Karuppayil 2014). EOs are oily substances produced by different parts of the plants, including flowers, buds, leaves, twigs, stems, seeds, and fruits (Bakkali et al. 2008). Generally, these oils are comprised of complex mixtures of volatile substances that are biosynthesized by plants. These substances can be broadly classified into several groups, such as aromatic and aliphatic compounds, terpenes, and terpenoids (Pichersky et al. 2006).

Most of the biological activities of EOs, particularly their antimicrobial activity, is associated with oxygenated terpenes, such as alcohols and phenolic terpenes. However, a few hydrocarbons have been found to exhibit significant antibacterial effects (Bassolé and Juliani 2012). Usually, the complex interactions between the diverse classes of phytoconstituents, such as phenols, alcohols, aldehydes, ketones, or other hydrocarbons of EOs are responsible for their antibacterial activities. In some cases, these interactions may lead to antagonistic or synergistic effects that contribute to the antibacterial activity of EOs, and even minor components of EOs can play a critical role in these effects (Gutierrez et al. 2008). It has been widely reported that EOs containing phenols or aldehydes, including thymol, eugenol, carvacrol, and cinnamaldehyde, as major components display higher antimicrobial activities than EOs containing terpenes or alcohols (Ait-Ouazzou et al. 2011; Sacchetti et al. 2005).

For instance, various species of *Thymus* and *Origanum* display excellent antimicrobial activities because of the presence of phenolic phytoconstituents including thymol and carvacrol (de Barros et al. 2009; Khan et al. 2018; Soković et al. 2009). In contrast, the high antibacterial activities of *Ocimum basilicum*, *Syzygium aromaticum*, and *Eugenia caryophillus* are attributed to eugenol (Vlase et al. 2014). Indeed, the EO of *Origanum* has been extensively studied because of its diverse contents and remarkable characteristics (Lukas et al. 2015). *Origanum* is an economically important genus belonging to the Lamiaceae family. Many genera, such as thyme (*Thymus*), sage (*Salvia*), lavender (*Lavandula*), basil (*Ocimum*), and mint (*Mentha*), of the Lamiaceae family are well known for their commercial values and their applications in ethnobotanical practices (Ibadullayeva et al. 2012). Within this family, *Origanum* is included in the subfamily Nepetoideae of tribe Mentheae and subtribe Menthinae and comprises about 40 species, which are naturally distributed in different parts of the world including the Mediterranean, Central Asia, the Arabian Peninsula, Northern Africa, and Europe (De Martino et al. 2009).

**Scheme 1:**
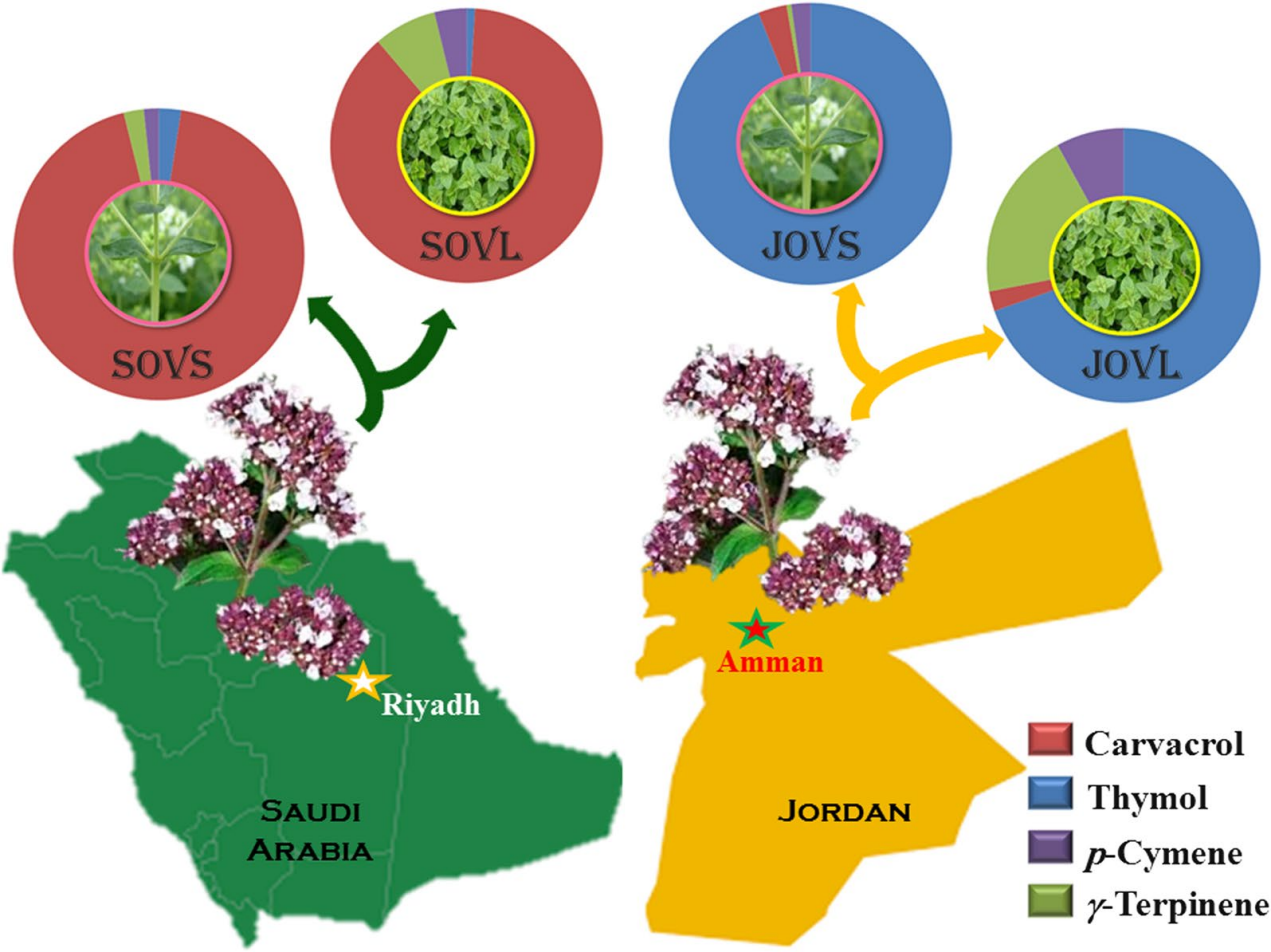
Composition of dominant components in the essential oils of Saudi and Jordanian *O. vulgare* L.

The *Origanum* genus is extensively found in the Mediterranean region, particularly concentrated in the eastern Mediterranean region (Aligiannis et al. 2001). These *Origanum* species are typically applied as flavoring agents for food but are also used as additives in some beverages (Janssen et al. 1987; Valnet et al. 1978). *Origanum* species are generally identified by the presence of a range of secondary metabolites and by the differences in the characteristic phytoconstituents of their essential oils. In particular, the EOs of *Origanum* species show great variation in their chemical diversity for various reasons, including ecological and environmental effects, as well as genetic variations (Vokou et al. 1993). In addition, other factors, including available nutrients (nitrogen, water, and minerals), photoperiod, radiation, and temperature also have a significant effect on the content and quality of the EOs of *O. vulgare* L. (Kokkini et al. 1994). Therefore, a comparative investigation of the EOs of *O. vulgare* L. from different regions would be useful to explore the chemical diversity of this species and to realize its industrial potential. Although extensive studies of the EOs of *Origanum* species have been conducted, however, *O. vulgare* L. populations from the Middle East have been poorly explored. Moreover, in majority of preveous studies, only the characterization of the oil composition of whole plant is described and determination of chemical components of different organs of *O. vulgare* L. and their comparison is very rare. Thus, in this study, we analyzed the phytochemical compounds of volatile oils extracted from the leaves and stems of *O. vulgare* L. grown in two Middle Eastern countries: Saudi Arabia and Jordan (Scheme 1). The chemical profiling of the EOs was performed by different characterization techniques including NMR, gas chromatography (GC)–flame ionization detector (FID), and GC–mass spectrometry (MS) techniques on two different (polar and non-polar) columns. Furthermore, the antimicrobial properties of EOs and their main compounds obtained from the plants of two different regions were also determined against Gram-negative and Gram-positive bacterial strains.

## Materials and methods

### Plant material

Whole plants of *O. vulgare* L. grown on the outskirts of Amman city in the north-central region of Jordan and in Al-Kharj, Saudi Arabia were procured in February and March of 2013, respectively. Verification of the plant materials was carried out by Dr. Jacob Thomas Pandalayil, a plant taxonomist at KSU, Riyadh. Representative samples of the plant species of Jordanian (OVHZK-303 J) and Saudi (OVHZK-303) *O. vulgare* L. are maintained in our research group laboratory.

### Isolation of volatile oils from the leaf and stem of *O. vulgare* L.

First, the leaves and stems of freshly harvested whole plants of *O. vulgare* L. were separated and cut into small pieces. The resultant pieces of the leaves and stems of *O. vulgare* L. grown in Jordan and Saudi Arabia were separately processed for hydro-distillation in a Clevenger apparatus for 3 h according to a previously reported method (Khan et al. 2018), yielding light-yellow oils. The yields of the oils from the leaves and stems of Saudi *O. vulgare* L. were 1.3% and 0.4% (w/w) on a fresh weight basis, respectively. The oil yields from the leaves and stems of Jordanian *O. vulgare* L. were 0.6% and 0.2% (w/w) on a fresh weight basis, respectively. The volatile oils attained after the hydro-distillation were dried using anhydrous Na_2_SO_4_ as the dehydrating agent and stored at 4 °C until further use.

### Chemicals

For the dilution of the essential oils, high purity diethyl ether bought from Sigma–Aldrich, Germany, was used. Pure essential oil components, e.g., carvacrol, *γ*-terpinene, *α*-pinene, thymol, and *β*-pinene, along with some essential oil fractions enriched with volatile components, such as camphene, *β*-caryophyllene, caryophyllene oxide, *cis*-3-hexen-1-ol, *p*-cymene, terpinene-4-ol, 1-octen-3-ol, *α*-terpinene, and 3-octanone, were available in our laboratory and were used for co-injection/comparative analysis.

### GC and GC–MS analysis of *O. vulgare* L. essential oils

The essential oils obtained through hydro-distillation of the leaves and stems of *O. vulgare* L. collected from Saudi Arabia and Jordan were analyzed on HP-5MS and DB-Wax columns using previously described methods (Khan et al. 2016). The identified constituents and the contents of the leaf and stem essential oils of Jordanian and Saudi *O. vulgare* L. are documented in Table 1 according to the elution order of each compounds on the HP-5MS column.

**Table 1:** Chemical constituents of leaf and stem volatile oils of *O. vulgare* L. grown in Saudi Arabia and Jordan

No.	Compound*	LRI_Lit_	LRI_Exp_^a^	LRI_Exp_^p^	SOVS (%)^b^	SOVL (%)^b^	JOVS (%)^b^	JOVL (%)^b^	Identification^c^
1	*trans*-2-Hexenal	846	852	1217	t	–	–	t	1,2
2	*cis*-3-Hexen-1-ol	850	854	1389	t	t	–	t	1,2,3
3	*trans*-2-Hexen-1-ol	854	857	1412	–	–	–	t	1,2
4	*cis*-2-hexen-1-ol	859	865	–	t	–	–	t	1,2
5	1-Hexanol	863	867	1358	–	–	–	t	1,2
6	2-Heptanol	894	897	–	t	t	–	–	1,2
7	Tricyclene	921	922	1010	–	t	–	t	1,2
*8*	*α-Thujene*	*924*	*927*	*1023*	*0.2 ± 0.00*	*1.4 ± 0.06*	*t*	*1.0 ± 0.51*	*1,2,3*
9	*α*-Pinene	932	934	1018	0.1	0.6	t	0.5	1,2,3
10	*α*-Fenchene	945	945	–	–	t	–	–	1,2
11	Camphene	946	949	1060	t	0.1	0.1	0.1	1,2,3
12	Benzaldehyde	952	961	1523	t	t	–	t	1,2
13	Sabinene	969	974	1118	0.1	0.6	–	0.1	1,2,3
14	*β*-Pinene	974	977	1104	t	0.3	–	0.1	1,2,3
15	1-Octen-3-ol	974	979	1455	0.2	0.4	0.8	1.2	1,2,3
16	3-Octanone	979	987	1255	t	t	0.1	0.1	1,2,3
17	6-Methyl-5-hepten-2-one^d^	981	–	1339	–	–	–	t	1,2
*18*	*β-Myrcene*	*988*	*992*	*1163*	*0.4 ± 0.05*	*2.0 ± 0.07*	*0.1 ± 0.00*	*1.9 ± 0.14*	*1,2,3*
*19*	*3-Octanol*	*988*	*996*	*1399*	*0.3*	*0.4*	*1.3*	*2.0*	*1,2,3*
20	*α*-Phellandrene	1002	1005	–	0.1	0.2	–	0.3	1,2
21	*δ*-3-Carene	1008	1011	1146	t	0.1	–	0.1	1,2
*22*	*α-Terpinene*	*1014*	*1017*	*1177*	*0.4 ± 0.00*	*1.4 ± 0.00*	*0.1 ± 0.00*	*2.7 ± 0.07*	*1,2,3*
23	*m*-Cymene	–	1023	–	–	t	–	–	1,2
*24*	*p-Cymene*	*1020*	*1025*	*1269*	*1.4 ± 0.07*	*3.2 ± 0.00*	*1.6 ± 0.07*	*6.8 ± 0.00*	*1,2,3*
25	Limonene^d^	1024	–	1196	0.1	0.3	–	0.4	1,2
26	*β*-Phellandrene	1025	1030	1205	0.2	0.5	0.1	0.5	1,2
27	1,8-Cineole	1026	1033	1208	t	t	–	t	1,2
28	*cis*-*β*-Ocimene	1032	1039	1235	t	0.1	–	0.2	1,2
29	Benzeneacetaldehyde	1036	1045	1635	t	t	–	t	1,2
30	*trans*-*β*-Ocimene	1044	1049	1252	t	0.1	–	0.1	1,2
*31*	*γ-Terpinene*	*1054*	*1060*	*1245*	*1.9 ± 0.06*	*6.2 ± 0.07*	*0.4 ± 0.00*	*17.0 ± 0.14*	*1,2,3*
32	*cis*-Sabinene hydrate	1065	1068	1471	0.6	0.9	0.3	0.3	1,2
33	*cis*-Linalool oxide^d^	1067	–	1048	–	–	–	t	1,2
34	*trans*-Linalool oxide	1084	1087	–	t	–	–	–	1,2
35	*α*-Terpinolene	1086	1090	1282	0.1	0.1	–	0.1	1,2,3
36	*p*-Cymenene^d^	1089	–	1438	–	–	–	t	1,2
37	Linalool^d^	1095	–	1552	0.1	0.2	0.6	t	1,2,3
*38*	*trans-Sabinene hydrate*	*1098*	*1099*	*1556*	*2.6 ± 0.08*	*3.5 ± 0.07*	*–*	*0.5 ± 0.03*	*1,2,3*
39	Nonanal	1100	1104	1394	–	–	–	t	1,2
40	*p*-Mentha-1(7), 8-diene^d^	1003	–	1167	–	–	–	t	1,2
41	1-Octen-3-yl acetate	1110	1113	–	t	–	–	–	1,2
42	1,3,8-*p*-Menthatriene	1108	1113	–	–	t	–	–	1,2
43	*cis*-*p*-Menth-2-en-1-ol	1118	1123	–	0.1	0.1	0.1	t	1,2
44	*α*-Campholenal	1122	1128	1491	t	t	–	–	1,2
45	*allo*-Ocimene	1128	1130	1373	–	t	–	t	1,2
46	*cis*-*p*-Mentha-2,8-dien-1-ol	1133	1138	–	–	t	–	–	1,2
47	*trans*-Pinocarveol	1135	1142	–	0.1	t	t	–	1,2
48	*trans*-Verbenol	1140	1148	1685	t	t	0.1	–	1,2
49	Isoborneol	1155	1159	–	–	t	–	–	1,2
50	Borneol	1165	1168	1708	0.7	0.1	1.4	0.2	1,2
51	Umbellulone	1167	1175	1646	t	t	–	–	1,2
*52*	*Terpinen-4-ol*	*1174*	*1180*	*1608*	*1.6 ± 0.06*	*0.9 ± 0.07*	*0.8 ± 0.00*	*0.5 ± 0.21*	*1,2,3*
53	*m*-Cymen-8-ol	1176	1184	–	t	–	–	–	1,2
54	*p*-Cymen-8-ol	1179	1188	1854	t	t	0.2	t	1,2
55	*α*-Terpineol	1186	1193	1703	0.1	0.1	0.2	t	1,2
56	Myrtenal	1195	1196	–	0.2	0.1	–	–	1,2
57	*cis*-Dihydro carvone	1191	1199	1611	0.1	0.2	0.1	0.1	1,2
58	*cis*-Piperitol^d^	1195	–	1753	t	t	–	–	1,2
59	*trans*-Dihydro carvone	1200	1203	1631	0.1	–	–	t	1,2
60	*n*-Decanal	1201	1207	1495	t	0.1	–	–	1,2
61	Verbenone	1204	1212	–	0.1	–	–	–	1,2
62	Linalool formate^d^	1214	–	1577	–	t	–	–	1,2
63	*trans*-Carveol	1215	1215	1842	t	t	–	t	1,2
64	*cis*-Carveol	1226	1229	–	t	t	0.1	–	1,2
65	Methyl thymol	1232	1233	–	t	t	–	–	1,2
66	Isobornyl formate	1235	1235	1584	–	t	–	t	1,2
67	(*E*)-Ocimenone	1235	1238	–	–	t	–	–	1,2
68	Methyl carvacrol	1241	1246	–	0.1	t	–	–	1,2
69	Carvotanacetone	1244	1249	1683	t	t	–	–	1,2
70	Geraniol	1249	1253	–	–	–	0.1	–	1,2
71	Linalool acetate	1254	1256	–	0.1	–	0.1	–	1,2
72	Thymoquinone	1248	1259	–	–	0.1		t	1,2
73	*cis*-Chrysanthenyl acetate	1261	1262	–	t	–	0.1	–	1,2
74	(2*E*)-Decenal	–	1266	–	–	t	–	–	1,2
75	Perilla aldehyde	1269	1281	–	t	–	–	–	1,2
76	Bornyl acetate	1284	1287	1585	t	t	–	t	1,2
*77*	*Thymol*	*1289*	*1294*	*2190*	*2.1 ± 0.00*	*0.8 ± 0.07*	*68.73 ± 4.50*	*59.1 ± 0.28*	*1,2,3,4*
*78*	*Carvacrol*	*1298*	*1311*	*2222*	*79.5 ± 0.77*	*72.8 ± 0.21*	*2.4 ± 0.14*	*2.0 ± 0.07*	*1,2,3,4*
79	(2*E*,4*E*)-Decadienal	1315	1319	–	t	0.1	–	–	1,2
80	Myrtenyl acetate	1324	1328	–	t	–	–	–	1,2
81	*trans*-Carvyl acetate	1339	1333		–	t	–	–	1,2
82	*δ*-Elemene	1335	1343	–	0.1	0.1	–	–	1,2
83	Thymol acetate	1349	1357	1870	t	t	–	t	1,2
84	Eugenol	1356	1361	2162	0.1	t	0.1	t	1,2
85	Carvacrol acetate	1370	1376	1876	0.2	0.1	–	t	1,2
86	*α*-Copaene	1374	1382	–	–	t		–	1,2
87	*β*-Bourbonene	1387	1391	–	–	t	t	–	1,2
88	*β*-Elemene	1389	1397	–	t	t	–	t	1,2
89	*n*-Tetradecane	1400	1400	–	t	t	–	–	1,2,3
90	Methyl eugenol	1403	1406	–	t	–	–	–	1,2
91	*cis*-*α*-Bergamotene	1411	1419	1570	t	t	t	–	1,2
*92*	*β-Caryophyllene*	*1417*	*1427*	*1600*	*1.5 ± 0.00*	*1.2 ± 0.00*	*2.5 ± 0.14*	*0.9 ± 0.00*	*1,2,3*
93	*β*-Copaene	1430	1436	–	t	t	–	–	1,2
94	*trans*-*α*-Bergamotene	1432	1440	1588	0.1	t	0.1	t	1,2
95	*α*-Guaiene	1437	1446	1592	t	t	–	–	1,2
96	Seychellene	1444	1453	–	–	t	–	–	1,2
97	*α*-Humulene	1452	1461	1672	0.1	0.1	0.1	t	1,2
98	*cis*-Muurola-4(14),5-diene	1465	1470	–	t	t	–	–	1,2
99	*γ*-Muurolene	1478	1483	1692	t	t	–	–	1,2
100	Germacrene-D	1484	1488	1712	0.2	0.2	0.1	t	1,2,3
101	*n*-Pentadecane	1500	1500	–	t	t	–	–	1,2,3
102	Bicyclogermacrene	1500	1504	1737	0.3	0.2	–	–	1,2,3
103	(*E*, *E*)-*α*-Farnesene	1505	1510	–	t	t	–	–	1,2
104	*β*-Bisabolene	1505	1513	1729	0.1	t	0.3	0.1	1,2
105	*β*-Ionol	1412	1517	1915	t	t	–	–	1,2
106	*γ*-Cadinene	1513	1521	1763	t	t	–	t	1,2
107	*trans*-Calamenene	1521	1529	1835	t	t	t	t	1,2
108	*α*-Cadinene	1537	1533	1777	t	–	–	–	1,2
109	*cis*-Nerolidol	1531	1547	2017	t	t	–	–	1,2
110	Thymohydro quinone	1553	1555	–	–	t	–	t	1,2
111	*trans*-Nerolidol	1561	1566	2046	t	–		0.1	1,2
112	Germacrene-d-4-ol	1574	1576	2057	t	t	–	–	1,2
113	Spathulenol	1577	1585	2132	0.1	t	–	–	1,2,3
114	Caryophyllene oxide	1582	1592	1991	0.1	t	0.3	0.1	1,2,3
115	Viridiflorol	1592	1600	–	t	t	–	–	1,2
116	Cedrol	1600	1610	–	–	–	–	t	1,2
117	Humulene epoxide II	1608	1614	–	t	–	–	–	1,2
118	1,10-*di*-*epi*-Cubenol	1618	1623	2065	t	t	–	–	1,2
119	*α*-Muurolol	1644	1646	–	–	t	–	t	1,2
120	*τ*-Cadinol	1638	1648	2179	0.1	–	–	–	1,2
121	*β*-Eudesmol	1649	1659	–	–	–	–	t	1,2
122	*α*-Cadinol	1652	1662	2237	t	t	0.t	–	1,2
123	*β*-Bisabolol	1674	1675	–	0.1	t	0.2	–	1,2
124	1-Tetradecanol	1671	1678	–	–	t	–	–	1,2
125	*α*-Bisabolol	1685	1689	–	–	–	t	–	1,2
126	*n*-Heptadecane	1700	1700	1700	t	t	–	–	1,2,3
127	Pentadecanal	–	1715	–	0.1	t	t	–	1,2
128	(*E, E*)-Farnesol	1742	1746	–	–	–	t	–	1,2
129	Tetradecanoic acid	–	1767	2693	–	–	0.1		1,2
130	14-Hydroxy-*α*-muurolene	1779	1780	–	t	–	–	–	1,2
131	Eudesm-7(11)-en-4-ol, acetate	1839	1846	–	t	–	t	–	1,2
132	Pentadecanoic acid	–	1871	–	t	t	t	–	1,2
133	*cis*-Spiroether	1879	1889	–	t	–	–	–	1,2
134	*trans*-Spiroether	1890	1896	–	t	t	–	–	1,2
135	2-Heptadecanone	–	1908	–	t	t	–	–	1,2
136	Methyl hexadecanoate	1921	1926	2208	t	–	–	–	1,2
137	Palmitic acid	1959	1959		t	–	0.3	–	1,2
138	*n*-Hexadecyl acetate	2003	2005	2301	0.1	t	0.2	–	1,2
139	(*E*, *E*)-Geranyl linalool	2026	2033	2541	0.7	t	0.9	–	1,2
140	Manool	2056	2068	2668	t	–	0.1	–	1,2
*141*	*3,3,4,5,5,8-hexamethyl-2,6-dihydro-s-indacene-1,7-dione*	*–*	*2083*	*2437*	*0.3 ± 0.00*	*0.1 ± 0.00*	*1.5 ± 0.00*	*–*	*1,2*
142	Phytol	1942	2105	2622	–	–	0.1	–	1,2
*143*	*2-tert-Butyl-4-(dimethylbenzyl)phenol*	*–*	*2125*	*2612*	*0.1 ± 0.05*	*t*	*2.1 ± 0.00*	*–*	*1,2*
144	Linoleic acid	2132	2141	–	0.2	t	1.2	–	1,2
145	Methyl octadecanoate	2124	2149	–	–	–	0.1	–	1,2
146	Octadecanoic acid	–	2179	–	–	–	0.1	–	1,2
147	*cis*-13-Octadecen-1-yl acetate	–	2193	–	t	–	0.1	–	1,2
148	*n*-Docosane	2200	2200	2200	0.1	t	0.2	–	1,2,3
149	*n*-Tricosane	2300	2300	2300	t	–	0.9	–	1,2,3
150	*trans*-Totarol	2314	2320	–	t	–	–	–	1,2
151	3*α*-Acetoxy manool	2359	2378	–	t	–	0.2	–	1,2
152	*n*-Pentacosane	2500	2500	2500	t	–	0.1	–	1,2,3
153	*n*-Hexacosane	2600	2600	2600	–	–	t	–	1,2,3
*Total identified*					*98.4*	*99.0*	*91.8*	*99.2*	

* Components are listed in their order of elution from HP-5 MS column; ^b^=Mean percentage calculated from flame ionization detector (FID) data and compounds higher than 1.0% are highlighted in italicface and their ± SD (n = 2) are mentioned; LRI_Lit_, linear retention index from the literature (Adams 2007); LRI_Exp_^a^, determined linear retention index against mixture of *n*-alkanes (C8-C31) on HP-5 MS column; LRI_Exp_^p^, determined linear retention index against mixture of *n*-alkanes (C8-C31) on DB-wax column; SOVS, stem volatile oils of Saudi *O. vulgare* L.; SOVL, leaves volatile oil of Saudi *O. vulgare* L.; *JOVS* stem volatile oils of Jordanian *O. vulgare* L.; *JOVL* leaves volatile oil of Jordanian *O. vulgare* L.; ^c^Identification by; 1, linear retention index (LRI) identical to literatures (*cf.* exp. part); 2, comparison of mass spectra (MS) with the library entries of mass spectra databases (*cf.* exp. part); 3, co-injection/comparison with the LRI and mass spectra of standards; 4, ^1^H and ^13^C NMR spectra; t, trace (<0.05%); ^d^Identified from DB-Wax column

### Retention indices

The linear retention indices (LRIs) of the leaf and stem essential oil compounds of Jordanian and Saudi *O. vulgare* L. were determined following a reported method (Khan et al. 2016), and these are listed in Table 1.

### Identification of volatile components

**Fig. 1:**
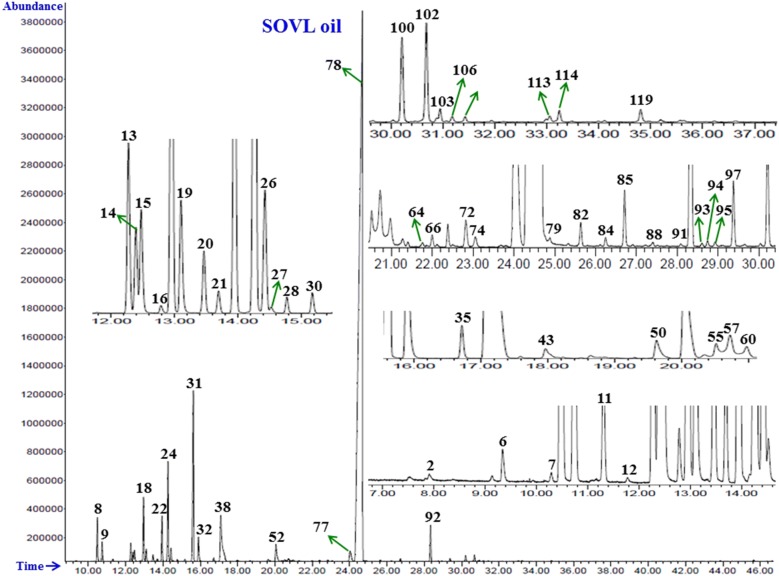
GC–FID chromatogram of essential oil from the leaves of Saudi *O. vulgare* L. obtained using an HP-5MS column. The characterized peaks are numbered according to the serial numbers in Table 1

**Fig. 2:**
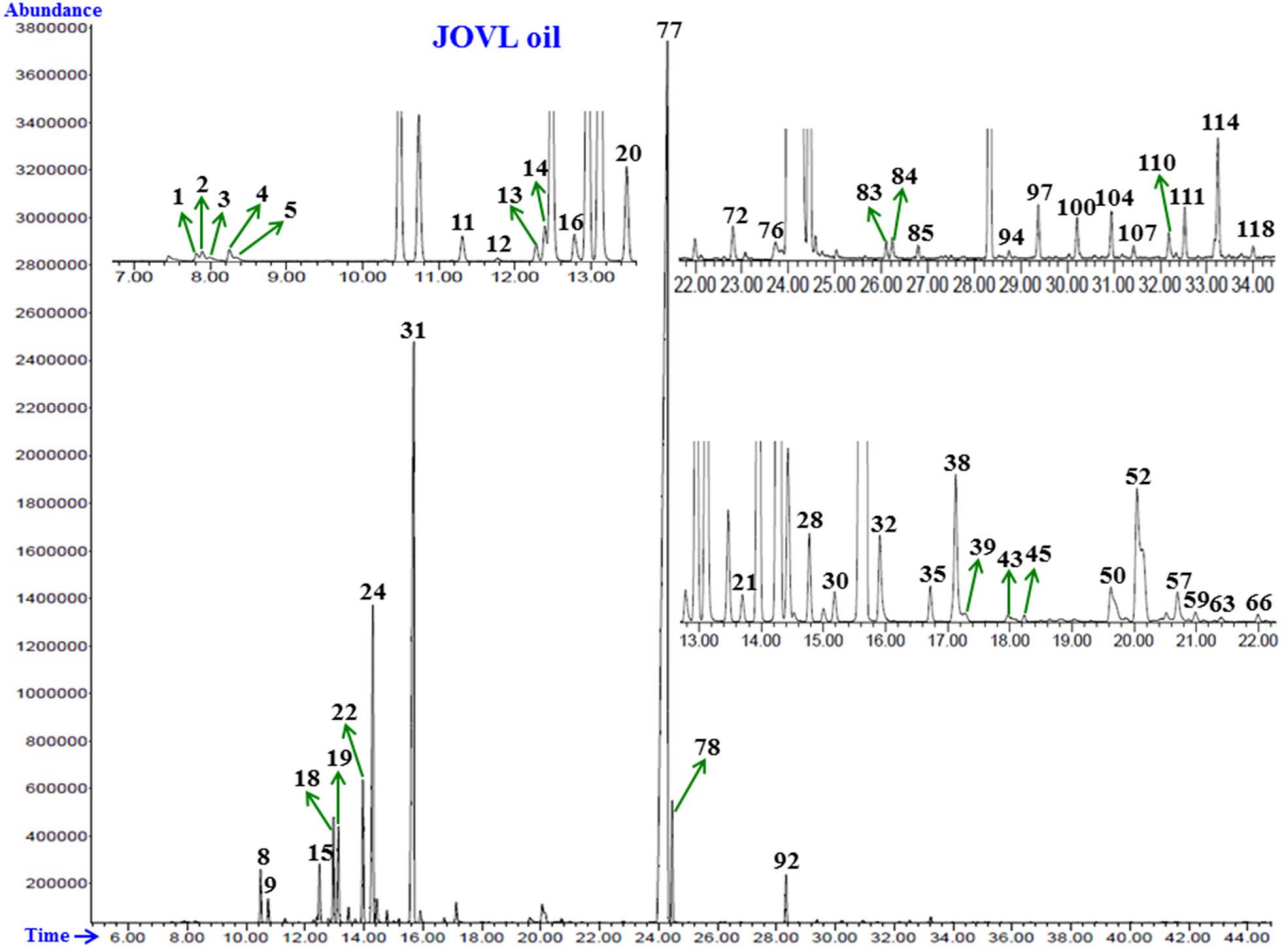
GC–FID chromatogram of essential oil from stems of Jordanian *O. vulgare* L. obtained using an HP-5MS column. The characterized peaks are numbered according to their serial numbers in Table 1

Identification of the volatile components was carried out via analysis on both columns (DB-Wax and HP-5MS) in a similar fashion to that reported previously (Khan et al. 2016). GC–FID Chromatogram with identified peaks of oil components on HP-5MS column is shown in Figs. 1, 2, Additional file 1: Figs. S1, S2.

### Nuclear magnetic resonance (NMR) analysis

The ^1^H and ^13^C NMR spectra of the pure compounds were done in similar fashion as described earlier (Khan et al. 2018). Details are given in supporting information (Additional file 1).

### Bacterial strains and growth medium

Four bacterial strains, *Escherichia coli* ATCC 25922, *Pseudomonas aeruginosa* ATCC 75853, *Micrococcus luteus* ATCC 10240, and *Staphylococcus aureus* ATCC 92213 were used as representative Gram-positive and Gram-negative bacteria. *E. coli*, *P. aeruginosa*, *M. luteus*, and *S. aureus* were grown on autoclaved Luria broth, nutrient broth, Müller–Hinton broth, and nutrient broth, respectively, at their respective optimal growth temperatures. For long-term preservation, strains were maintained on agar plates of their respective media and were stored at − 80 °C in 20% glycerol.

### Evaluation of antimicrobial activity

For determining the antimicrobial activity of the test compounds, microdilution assays in 96-well plates were used. Cells of *E. coli*, *P. aeruginosa*, *M. luteus*, and *S. aureus* were grown in their respective broths until the logarithmic growth phase. An aliquot of 10 µL from each culture was added to each well of a 96-well plate containing 90 µL of fresh culture medium. Test compounds prepared in dimethyl sulfoxide (DMSO) were added to the wells in triplicate to final concentrations of 50, 100, 200, 300, 400, and 500 µg/mL. Ampicillin (Amp) and kanamycin (Km) were added to final concentrations of 10, 20, 30, 40, and 50 µg/mL to the culture media as positive controls. The plates were incubated on a rotary shaker at 37 °C and 140 rpm for 8 h. The optical absorbance at 600 nm (OD600) was measured using an enzyme-linked immunosorbent assay reader (Multiskan Ascent, Labsystems, Helsinki, Finland) at hourly intervals. The OD600 at a given time was subtracted from the OD600 at 0 h to record the change in the OD of each sample. The results presented are the mean ± standard error of three independent experiments. P values were calculated using an unpaired Student’s *t*-test in GraphPad (GraphPad Software, Inc., La Jolla, CA, USA). The p values considered significant for different tests are mentioned in the figure captions. MIC and IC_50_ values were calculated using the standard protocols and have been described elsewhere (Khan et al. 2017; Veiga et al. 2019).

## Results

The hydro-distillation of the leaves and stems of *O. vulgare* L. from both Saudi Arabia and Jordan was performed in a Clevenger-type apparatus, which yielded light-yellow oils. Based on the fresh weight of the materials, the EOs of the leaves and stems of Saudi *O. vulgare* L. were obtained in yields of 1.30% and 0.40% w/w; in contrast, the EOs of the same parts of its Jordanian counterpart were found to be 0.60% and 0.24% w/w from the leaves and stems, respectively. Notably, the studied parts of both Saudi and Jordanian *O. vulgare* L. produced a good oil yield, ranging from 0.2 to 1.3%, when compared to their counterparts found in different parts of the world, as shown in Additional file 1: Table S1. Apart from the *Origanum* species found in Turkey and Tunisia, which produce an excellent oil yield in the range of 4–7%, most species found in other parts of the world produce oil yields of < 1% (Additional file 1: Table S1).

A detailed phytochemical analysis of the essential oils led to the identification of a total of 153 compounds from these oils (EOs from the leaves and stems of *O. vulgare* L. from Saudi Arabia and Jordan). The analysis was performed via GC–MS and GC–FID using both polar and nonpolar columns. Among these constituents, 28 compounds were found to be common in the EOs of the leaves and stems of *O. vulgare* L. from both regions. Notably, compounds 13 and 15 are specific to the EOs of the leaves and stems, respectively, of Saudi *O. vulgare* L., whereas compounds 9 and 8 were only found in the leaves and stems, respectively, of Jordanian *O. vulgare* L. The identified compounds and their relative contents are listed in Table 1 according to their elution order on a nonpolar HP-5MS column.

**Fig. 3:**
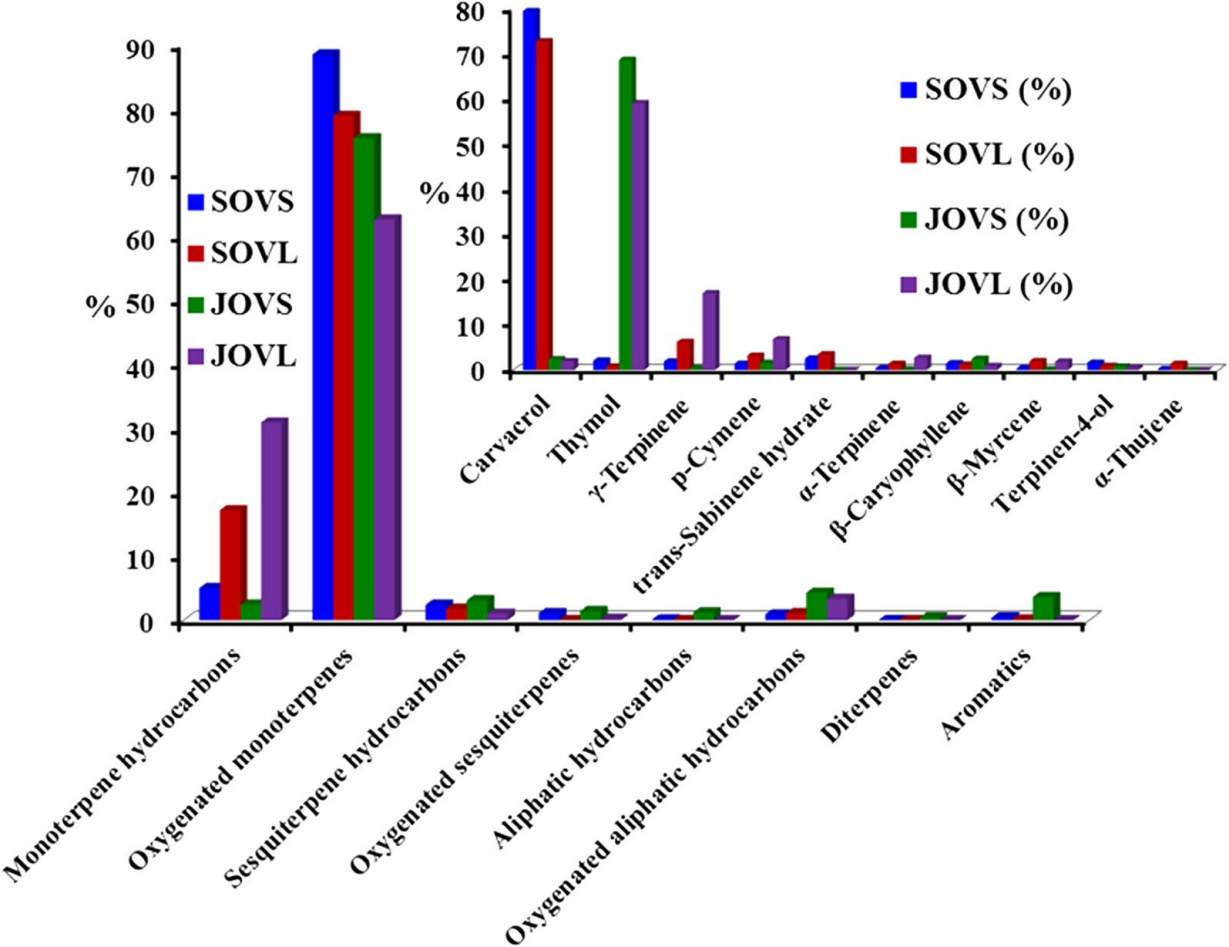
Content of major compounds and chemical classes in the leaf and stem essential oils of Saudi and Jordanian *O. vulgare* L.

The phytochemical constituents of the stems and leaves of both Saudi and Jordanian *O. vulgare* L. samples are dominated by oxygenated monoterpenes. Among the studied EOs, the stem and leaf oils of the Saudi plant contain the largest amount of monoterpenes, i.e., 88.5% and 78.9%, respectively, whereas its Jordanian counterpart contains 75.4% and 62.7%, respectively. The next major chemical class is the monoterpene hydrocarbons, which are present in large amount in the leaf oils of both Saudi (17.2%) and Jordanian (30.9%) plants, where their stem oils contain 5.0% and 2.4%, respectively. The other constituents, which were found in relatively smaller amounts in all studied EOs, are sesquiterpene hydrocarbons, oxygenated sesquiterpenes, aliphatic hydrocarbons, oxygenated aliphatic hydrocarbons, aromatics, and diterpenes (Fig. 3). Totally, 69 monoterpenoids were identified in the studied EOs. The oxygenated monoterpenes are mainly comprised of thymol, carvacrol, *trans*-sabinene hydrate, and terpinen-4-ol. Among the squiterpenoids, only *β*-caryophyllene was found in considerable quantity, whereas the other sesquiterpenoids such as germacrene D, germacrene D-4-ol, spathulenol, and caryophyllene oxide were present in minute concentrations (cf. Table 1).

### Antimicrobial activity

**Fig. 4:**
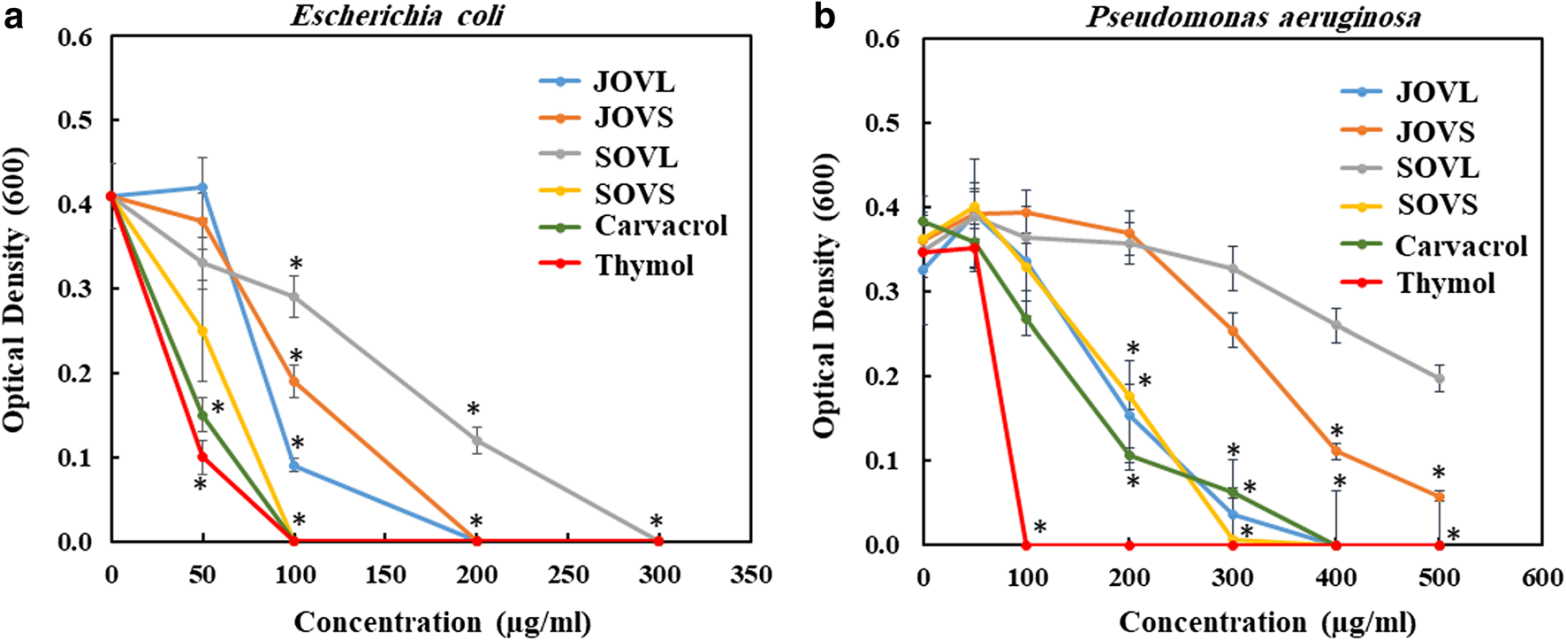
Change in OD_600_ of Gram-negative bacteria (*E. coli* (**a**) and *P. aeruginosa* (**b**) following treatment with different samples. Asterisk indicates the values that are significantly different from the control (*p* value < 0.005)

All the tested samples exhibited significant antimicrobial activity against both Gram-positive and Gram-negative bacteria. The growth inhibition of *E. coli* (a commonly used Gram-negative bacteria), which was measured in terms of the change in OD600 with various concentrations of the test compounds, is shown in Fig. 4a.

Clearly, Saudi *O. vulgaris* leaf EO (SOVL) was the least inhibitory, and the complete inhibition of *E. coli* growth was observed only at a concentration of 300 µg/mL. Thymol exhibited the highest antimicrobial activity, inhibiting the growth of *E. coli* completely at a concentration of 100 µg/mL (Fig. 4). The antimicrobial activities of carvacrol and Saudi *O. vulgare* L. stem EO (SOVS) were comparable to those of thymol but varied in their half maximal inhibitory concentration (IC_50_) values, as listed in Table 2. In contrast, Jordanian *O. vulgare* L. leaf and stem EOs (JOVL and JOVS, respectively) inhibited the growth of *E. coli* completely at a concentration of 200 µg/mL.

**Table 2:** The IC_50_ values obtained with different samples against Gram-negative and Gram-positive bacteria

Organism	IC_50_ (µg/mL)							
	JOVL	JOVS	SOVL	SOVS	Carvacrol	Thymol	Amp^a^	Km^a^
Gram-negative								
*E. coli*	99	107	150	55	54	43	40	10
*P. aeruginosa*	190	325	430	196	151	63	20	40
Gram-positive								
*M. luteus*	84	77	800	67	66	27	20	40
*S*. *aureus*	77	83	380	63	53	41	250	15

^a^MIC values obatined in this study

The antimicrobial activity of the samples against another Gram-negative bacterium, *P. aeruginosa*, is shown in Fig. 4b. Because *P. aeruginosa* grows more vigorously than *E. coli*, inhibition was observed at a comparatively higher concentration. As observed for *E. coli*, the most effective compound against *P. aeruginosa* was also thymol, inhibiting growth completely at a concentration of 100 µg/mL. In contrast, carvacrol inhibited growth at 300 µg/mL. The SOVL could not inhibit the growth completely, even at the highest test concentration, 500 µg/mL. The minimum inhibitory concentration (MIC) values of the tested samples against *P. aeruginosa* are listed in Table 2. Based on these values, the compounds can be arranged in order according to their microbicidal activity against *P. aeruginosa* (Table 3).

**Table 3:** Antimicrobial activity of essential oils and their purified compounds

Bacteria type	Organisms	Activity
Gram-negative	*E. coli*	Thymol>carvacrol>SOVS>JOVL>JOVS>SOVL
	*P. aeruginosa*	Thymol>carvacrol>JOVL>SOVS>JOVS>SOVL
Gram-positive	*M. luteus*	Thymol>carvacrol>JOVL>SOVS>JOVL>SOVL
	*S*. *aureus*	Thymol>carvacrol>SOVS>JOVL>JOVS>SOVL

**Fig. 5:**
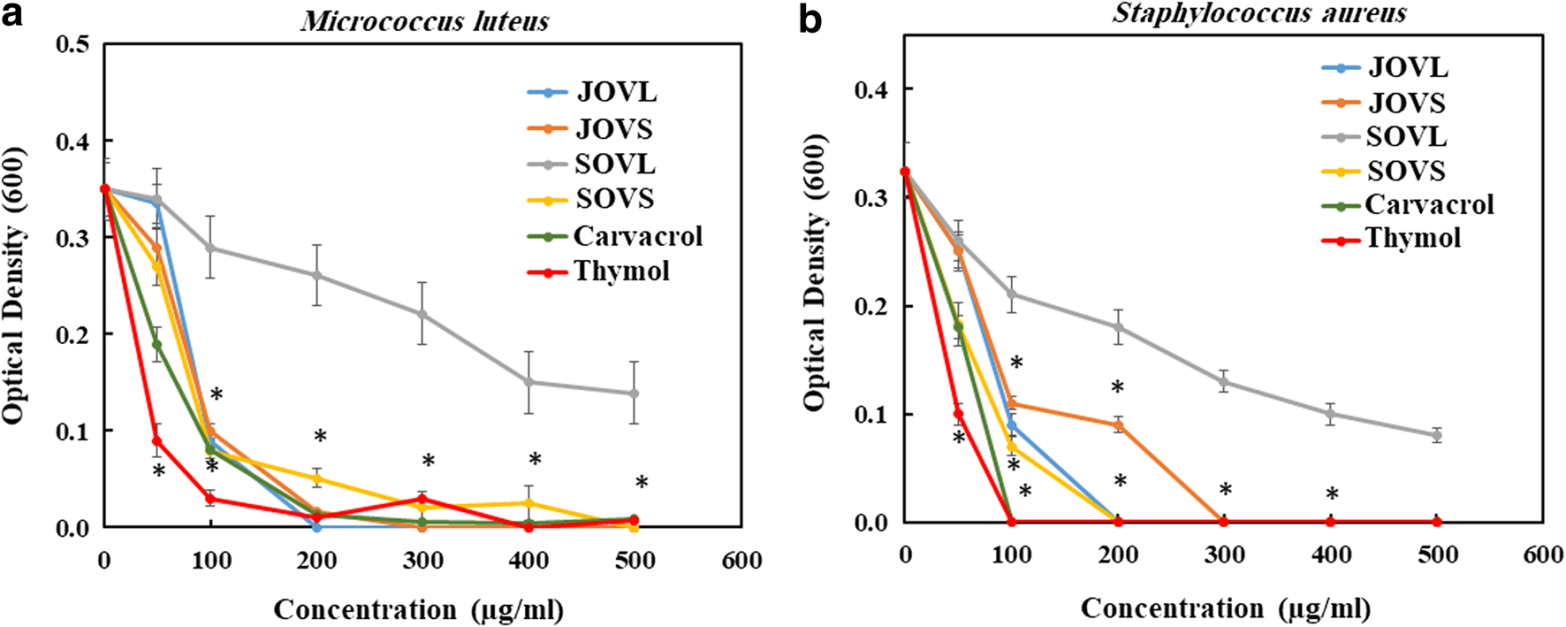
Growth inhibition of Gram-positive bacteria (*M. luteus* (**a**) and *S. aureus* (**b**)), as measured by the change in OD_600_ following treatment with different samples. Asterisk indicates the values that are significantly different from the control (*p* value < 0.005)

All the tested samples also exhibited good antimicrobial activity against the two tested Gram-positive bacteria. The antimicrobial activity of the tested samples against *M. luteus* is shown in Fig. 5. The figure clearly shows that SOVL was the least effective in inhibiting the growth of *M. luteus*, and complete growth inhibition was not observed even at the highest test concentration of 500 µg/mL. However, thymol most effectively inhibited the growth of *M. luteus*, showing significant growth inhibition at 50 µg/mL (Fig. 5a). In contrast, carvacrol was able to completely inhibit the growth of *M. luteus* only at 100 µg/mL. Based on the IC_50_ values, the antimicrobial activity against *M. luteus* can be arranged in the order given in Table 3.

A similar trend in the antimicrobial activity of the samples was observed against another Gram-positive bacteria, *S. aureus* (Fig. 5b). In this test, thymol most effectively inhibited the growth of *S. aureus*, and SOVL was the least effective against *S. aureus*. Hence, the test compounds can be arranged in the order given in Table 3 based on their IC_50_ values against *S. aureus*.

The trend in antimicrobial activity was very similar in all the four tested bacteria. However, it is evident from the MIC values listed in Table 2 that the tested compounds exhibited higher antimicrobial activity against the tested Gram-positive bacteria than against the Gram-negative bacteria. This trend is also in agreement with most of the studies investigating the action of whole EOs against food spoilage organisms and foodborne pathogens, which suggest that the EOs are slightly more active against Gram-positive than Gram-negative bacteria (Burt 2004). However, in several studies, the reverse trend has been observed (Pesavento et al. 2015). Typically, Gram-negative bacteria have an outer membrane surrounding the cell wall, which makes them susceptible to the action of common antibacterial agents (Vaara 1992). Notably, the MIC values of the samples tested in this study were many times lower than the MIC values of kanamycin and ampicillin obtained in this study (Table 2).

## Discussion

Typically, the essential oils of plants of the same species grown in different locations exhibit significant variations in composition because of the different environmental conditions, such as altitude, solar exposure, and soil composition (Figueiredo et al. 2008). These geographic variations of the yield and composition of volatile oils have been found in several species, demonstrating that distinct chemotypes of plants grow in different locations (Bhatt et al. 2019; Fikry et al. 2019; Hussain et al. 2008; Tanasescu et al. 2019). Therefore, the study of the chemical variability and yield of volatile oils of commercially important plants such as *O. vulgare* L. grown in different locations is highly desirable. In this study, we made a detailed analysis of the chemical constituents of the leaf and stem volatile oils of *O. vulgare* L. grown in two different geographical locations: Saudi Arabia and Jordan. This study is the first example of the characterization of the essential oil constituents of the leaf and stem volatile oils of *O. vulgare* L. from these regions, and the components are compared with the EOs of *O. vulgare* L. grown in other parts of the world.

Based on the monoterpene constituents, both Saudi and Jordanian plants are classified as cymyl chemotypes, which is typical of various *Origanum* species grown in the Mediterranean (Lukas et al. 2015). For instance, the *Origanum* populations of southern and coastal Europe including Portugal, Spain, and Greece are often dominated by the cymyl chemotype. The *Origanum* species from the Middle East have been rarely studied, but the results obtained from the essential oils of Saudi and Jordanian *O. vulgare* L. indicate that the cymyl chemotype should predominate in most *Origanum* species from these regions. Althogh studied oils are classified as cymyl chemotypes, however, significant quantitative differences between four different oils are clearly apparent in two major isomeric phenols, i.e., carvacrol and thymol, and their biosynthetic precursors, i.e., *γ*-terpinene and *p*-cymene (Sivropoulou et al. 1996). For instance, the stem and leaf oils of Saudi *Origanum* contain carvacrol as the major component, containing 79.5% and 71.9%, respectively, followed by *γ*-terpinene (stem oil 1.9% and leaf oil 6.2%) and *p*-cymene (stem oil 1.4% and leaf oil 3.2%); thus, it is characterized as a carvacrol chemotype. In contrast, the studied oils of Jordanian *Origanum* contain thymol as the major component, containing 68.7% in the stem oil and 59.1% in the leaf oil, while its biosynthetic precursors *γ*-terpinene (stem oil 0.4% and leaf oil 17.0%) and *p*-cymene (stem oil 1.6% and leaf oil 6.8%) were also present in significant amounts; therefore, it is characterized as a thymol chemotype (Fig. 3). This variation in the phytochemical constituents of the stem and leaf oils of both Jordanian and Saudi *O. vulgare* L. can be attributed to the differences in the climatic conditions, geographical location of collection sites, and other genetic factors, as has been observed in several other species of *Origanum* from different regions (Sarikurkcu et al. 2015; Vokou et al. 1993).

Typically, the formation of thymol and carvacrol involves the hydroxylation of *γ*-terpinene and *p*-cymene precursors (Poulose and Croteau 1978). This process involves cytochrome P450 monooxygenases for the conversion of *γ*-terpinene to thymol and carvacrol via eleven cytochrome P450 gene sequences (CYP71D178-CYP71D182) from oregano, thyme, and marjoram (Crocoll et al. 2010). Thus, it has been suggested that CYP71D179/182 is responsible for the biosynthesis of thymol, whereas CYP71D181 may be involved in carvacrol biosynthesis. Therefore, in this study, the presence of a large amount of thymol in Jordanian *Origanum* can be attributed to the aforementioned biosynthetic process, in which CYP71D179/182 transcription of P450 is more active compared to the transcription of other genes. In contrast, CYP71D181 transcription might play a more active role in the biosynthesis of carvacrol in Saudi *Origanum*. Therefore, apart from the climatic and geographic conditions, other enzymatic processes may also be responsible for the variation in the phytochemical constituents of both Saudi and Jordanian *Origanum* plants.

The phytoconstituents of the studied EOs were further identified by advanced characterization techniques, including ^1^H and ^13^C NMR (cf. Additional file 1: Fig. S3a, b). In these constituents, thymol (2-isopropyl-5-methylphenol), and its isomer carvacrol (2-methyl-5-(1-methylethyl)-phenol) were identified as the major components of Jordanian and Saudi *Origanum* respectively. These isomeric phytomolecules have widespread applications in various fields including pharmaceutical, food and cosmetic industries (Javed et al. 2013; Sobczak et al. 2014; Venturini et al. 2002; Andersen 2006; Suntres et al. 2015).

In this study, we found that Jordanian *Origanum* is an important source of thymol, whereas carvacrol can be obtained on a large scale from Saudi *Origanum*. Apart from thymol and carvacrol (Additional file 1: Fig. S4), some of the other phytochemical components such as, *α*-thujene, *β*-myrcene, *α*-terpinene, *m*-cymene, *p*-cymene, *γ*-terpinene, *trans*-sabinene hydrate, terpinen-4-ol, *β*-caryophyllene, 3,3,4,5,5,8-hexamethyl-2,6-dihydro-s-indacene-1,7-dione, and 2-tert-butyl-4-(dimethylbenzyl)phenol were found in noteworthy amounts in the studied oils. The stem and leaf EOs of Jordanian *Origanum* show several qualitative similarities, which is clearly reflected by the presence of 28 components in both samples in considerable amounts, although their relative quantities are different, i.e., *β*-myrcene (0.1% and 1.9%), *α*-terpinene (0.1% and 2.7%), *p*-cymene (1.6% and 6.8%), *γ*-terpinene (0.4% and 17.0%), terpinen-4-ol (0.8% and 0.5%), and *β*-caryophyllene (2.5% and 0.9%), respectively. In addition, certain components could be found in only one of the oils; for example, 3,3,4,5,5,8-hexamethyl-2,6-dihydro-s-indacene-1,7-dione (1.5%) and 2-*tert*-butyl-4-(dimethylbenzyl)phenol (2.1%) (Additional file 1: Fig. S4) were only present in the stem oil of Jordanian *Origanum*. Notably, the leaf oil of Jordanian *Origanum* proved to be an excellent source of *γ*-terpinene, which is present in large amounts (17.0%) in the sample. Although the Saudi *Origanum* contain similar phytochemical constituents in different quantities, as shown in Table 1, certain components are specific to this particular species. For instance, *trans*-sabinene hydrate, 2-heptanol, *α*-thujene, *α*-campholenal, *cis*-*p*-mentha-2,8-dien-1-ol, *iso*borneol, umbellulone, *m*-cymen-8-ol, myrtenal, *cis*-piperitol, *n*-decanal, methyl carvacrol, and carvacrol acetate are present in minute concentrations or in trace amounts.

A correlation between the antimicrobial activity of the tested compounds and the compositions of the stem and leaf oils of both Saudi and Jordanian origin used in this study was made. On comparing the activities of these compounds against the four organisms studied, it was found that thymol has showed the highest activity against all the tested organisms, followed by that of carvacrol. Both thymol and carvacrol are structurally very similar, having the hydroxyl group at a different location on the phenol ring. Typically, they appear to act by making the cell membrane permeable. In the case of Gram-negative bacteria, thymol and carvacrol induce the disintegration of the outer membrane, releasing lipopolysaccharides (LPS) and increasing the permeability of the cytoplasmic membrane to adenosine triphosphate (ATP) (Burt 2004). Furthermore, it has been proposed that these compounds can interact with membrane proteins and enzymes, as well as intracellular targets (Engel et al. 2017). In most cases, both thymol and carvacrol exhibit comparable antimicrobial properties because of their similar structures. However, in some cases, thymol has demonstrated better activity compared to carvacrol under similar conditions.

Studies of the antibacterial activity of thymol and carvacrol isolated from the EO of *O. dictamnus* L. have revealed that thymol exhibits stronger activity than carvacrol against most microbial types (Liolios et al. 2009). Similarly, in our previous study on *S. mutans* (a well-known oral pathogen), we observed that thymol showed a relatively higher activity than carvacrol. Thymol showed higher activity by effectively inhibiting the growth of the tested organism by inducing stress and autolysis (Khan et al. 2017). Thymol also significantly disrupts the biofilms formed by *S. mutans*. As far as SOVL, SOVS, JOVL, and JOVS are concerned, JOVL and SOVS exhibited the highest antimicrobial activities against all the four tested organisms. Furthermore, the antimicrobial activities of these two essential oils were comparable. As shown in Table 1, JOVL contains 59.1% thymol and 2.0% carvacrol, while SOVS contains about 79.5% carvacrol and 2.1% thymol. Therefore, SOVS has the highest amount of the pure compound probably responsible for the remarkable antimicrobial activity. The cumulative effect of carvacrol and thymol has not yet been examined. JOVL shows significant activity, probably because of the cumulative effect of thymol with some other constituents including carvacrol and γ-terpinene. Notably, JOVL consists of the highest amount of γ-terpinene, constituting 17.0% of the total. γ-Terpinene is already known to have significant antimicrobial activity against a number of pathogenic microorganisms, even at a concentration of 0.1% (v/v) (Delaquis et al. 2002). JOVL also contains 7.0% cymene (alkyl benzene), which has also been shown to have good antimicrobial activity (Delaquis et al. 2002). Thus, it is very highly likely that the cumulative effect of thymol, carvacrol, *p*-cymene, and *γ*-terpinene resulted in the remarkable antimicrobial activity that was observed in this study.

The next most active essential oil was JOVS, which contains 68.7% thymol and 2.4% carvacrol. Other constituents that were present in significant amount in this essential oil were *β*-caryophyllene (2.5%), 2-*tert*-butyl-4-(dimethylbenzyl) phenol (2.1%), and *p*-cymene (1.6%). The cumulative effect of all these compounds may be responsible for the antimicrobial activity. The least activity was observed for SOVL, which mainly contain carvacrol (72.8%), *γ*-terpinene (6.2%), *trans*-sabinene hydrate (3.5%), *p*-cymene (3.2%), and *β*-myrcene (2.0%). These results reveal that the whole EOs and individual components of the oils studied, including thymol and carvacrol, show different degrees of activity against Gram-positive and Gram-negative bacteria. This is in agreement with the trend that the chemical composition of the EOs from a particular plant species vary with geographical origin and harvesting period. However, we can only speculate about the effects of the constituents and their combinations on the antimicrobial activity, and future detailed investigations on antibiofilm activity and antiquoroum sensing activities are required. Based on our results, we propose that plant species from different regions produce different compounds or mixtures of the compounds, and these variations result in completely different bioactivities.

## Supplementary information

**Additional file 1.** Additional figures and tables.

## Data Availability

The [GC–MS/GC–FID, NMR] data used to support the findings of this study are included within the article and additional file(s).
