# Supplementary material for: Chemical diversity in leaf and stem essential oils of Origanum vulgare L. and their effects on microbicidal activities
Source: AMB Express. 2019 Oct 31;9:176. doi: 10.1186/s13568-019-0893-3 (PMC6823331; doi:10.1186/s13568-019-0893-3)
Supplement: Supplementary file 1 — Additional file 1. Additional figures and tables. [file 13568_2019_893_MOESM1_ESM.docx]

**Chemical diversity in leaf and stem essential oils of *Origanum vulgare* L. and their effects on microbicidal activities**

Merajuddin Khan,*^a^ Shams Tabrez Khan,^b^ Mujeeb Khan,^a^ Ahmad A. Mousa,^a^ Adeem Mahmood,^a^ and Hamad Z. Alkhathlan*^a^

*^a^Department of Chemistry, College of Science, King Saud University, P.O. Box 2455 , Riyadh - 11451, Saudi Arabia*

*^b^Department of Agricultural Microbiology, Aligarh Muslim University, Aligarh 202002, U.P., India*

*Corresponding Author: *E-mail:* [mkhan3@ksu.edu.sa](mailto:mkhan3@ksu.edu.sa) (M. Khan); [khathlan@ksu.edu.sa](mailto:khathlan@ksu.edu.sa) (H.Z. Alkhathlan)

**Table S1.** Oil yield of *O. vulgare* L. from different parts of the world

| **Geographical regions** | **Major components (%)** | **Oil yield (%)** | **Reference** |
| --- | --- | --- | --- |
| **Turkey** | | | |
| Edirne (subsp. *vulgare*) | Thymol (58.3), carvacrol (16.1), *p*-cymene (13.5) and *γ*-terpinene (4.5). | 5.1^a^ (w/dw) | ([Sarikurkcu et al. 2015](#_ENREF_47)) |
| Karaman (subsp. *hirtum*) | Linalool (96.3^a^). | 7.3^a^ (w/dw) | ([Sarikurkcu et al. 2015](#_ENREF_47)) |
| Aegean (subsp. *hirtum*) | Carvacrol (23.43–78.73). | 1.3-6.5^n^ (dw^d^) | ([Baser et al. 1994](#_ENREF_9)) |
| Erzurum (subsp. vulgare) | Caryophyllene (14.4), spathulenol (11.6), germacrene-D (8.1), α-terpineol (7.5), caryophyllene oxide (5.8). | 2.3a (v/dw) | ([Şahin et al. 2004](#_ENREF_46)) |
| **Tunisia** | | | |
| Nefza(subsp.g*landulosum*) | Thymol (31.8-46.1), carvacrol (1.7-2.9), *p*-cymene (11.5-35.7) and *γ*-terpinene (24.0-27.1). | 4.3-5.8^b^ (v/dw) | ([Mechergui et al. 2016](#_ENREF_38)) |
| Krib (subsp. *glandulosum*) | Thymol (18.4-31.5), carvacrol (7.7-15.1), *p*-cymene (27.3-46.3) and *γ*-terpinene (16.1-23.5). | 2.7-3.4^b^ (v/dw) | ([Mechergui et al. 2016](#_ENREF_38)) |
| **China** | | | |
| Kunlun Mountain of Hetian | *β*-Citronellol (85.3), citronellol acetate (5.2). | 0.7^w^ (w/dw) | ([Gong et al. 2014](#_ENREF_20)) |
| Shangqiu of Henan | Thymol (42.9), citronellol (12.2), *β*-caryophyllene (7.8), *p*-cymen-2-ol (7.5), *m*-cymene (7.4). | 0.3^w^ (w/dw) | ([Gong et al. 2014](#_ENREF_20)) |
| Hetian | *β*-Citronellol (75.0), (*E*)-geraniol (7.7). | 0.3^w^ (w/dw) | ([Gong et al. 2014](#_ENREF_20)) |
| Anhui | 1,8-Cineole (20.8), *β*-carvophyllene (10.2), eugenol methyl ether (9.8), citronellol (8.8), *β*-linalool (5.5). | 0.3^w^ (w/dw) | ([Gong et al. 2014](#_ENREF_20)) |
| Yili | Caryophyllene oxide (32.9), *β*-carvophyllene (17.7), citronellol (10.2), germacrene D (9.8), *β*-bisabolene (6.8), *α*-humulene (5.6). | 0.1^w^ (w/dw) | ([Gong et al. 2014](#_ENREF_20)) |
| **Pakistan** | *β*-Citronellol (72.7), thymol (7.2), citronellol acetate (5.2). | 0.3^w^ (w/dw) | ([Gong et al. 2014](#_ENREF_20)) |
| **Iran** | | | |
| Noshahr (subsp. *vulgare*) | Thymol (37.1), *γ*-terpinene (9.7), carvacrol (9.6), carvacrol methyl ether (6.9), *cis*-*α*-bisabolene (6.8). | 0.5^a^ (v/dw) | ([Vazirian et al. 2015](#_ENREF_54)) |
| Zardavan (subsp. *Gracile*) | Carvacrol (46.5), *γ*-terpinene (13.9), *p*-cymene. (13.5), Carvacrol methyl ether (7.2). | 1.4^l^ (dw^d^) | ([Moradi et al. 2015](#_ENREF_39)) |
| Zardavan (subsp. *Gracile*) | Carvacrol (60.6), γ-terpinene (16.6), *p*-cymene (7.2). | 2.4^f^ (dw^d^) | ([Moradi et al. 2015](#_ENREF_39)) |
| **Italy** | | | |
| Marconia di Pisticci (subsp. *hirtum*) | Thymol and carvacrol (84.7), *trans*-caryophyllene (1.5). | 2.7^a^ (v/fw) | ([Mancini et al. 2014](#_ENREF_37)) |
| Mandia (subsp. *hirtum*) | Thymol and carvacrol (75.1), germacrene A (2.0), *trans*-caryophyllene (2.7). | 1.0^a^ (v/fw) | ([Mancini et al. 2014](#_ENREF_37)) |
| San Giovanni a Piro (subsp. *hirtum*) | Thymol and carvacrol (65.3), germacrene A (4.0), *trans*-caryophyllene (3.2). | 1.0^a^ (v/fw) | ([Mancini et al. 2014](#_ENREF_37)) |
| **India** | | | |
| Purara | Thymol (27.9), *p*-cymene (25.1), *γ*-terpinene (7.9), and 1 octen-3-ol (4.7). | 0.4^l^ (v/fw) | ([Verma et al. 2012](#_ENREF_56)) |
| Purara | Thymol (49.0), *p*-cymene (13.9), γ-terpinene (8.7), and 1 octen-3-ol (1.8). | 0.7^f^ (v/fw) | ([Verma et al. 2012](#_ENREF_56)) |
| **Corsica** | Carvacrol (0.6-65.5), thymol (0.0-49.5), sabinene (7.8-20.2), *cis*-sabinene hydrate (0.7-24.8), *cis*-sabinene hydrate (0.0-52.4), *γ*-terpinene (0.6-35.4). | 0.1-1.8^a^ (w/dw) | ([Lukas et al. 2008](#_ENREF_34)) |
| **Poland** (subsp. *vulgare*) | Sabinene (10.9-25.5), germacrene D (9.36-15.3), *Z*-(*β*)-ocimene (9.10-16.3), *E*-caryophyllene (9.4-12.9). | 0.7-0.9^n^ (dw^d^) | ([Nurzyńska-Wierdak et al. 2012](#_ENREF_40)) |
| **Estonia** (subsp. *vulgare*) | Linalool (0.3-20.6), *β*-caryophyllene (1.3-45.0), germacrene D (0.7-21.0), caryophyllene oxide (1.5-31.3) and spathulenol (0.9-10.1). | 0.2-0.4^n^ (dw^d^) | ([Ivask et al. 2005](#_ENREF_24)) |
| **Bulgaria** (subsp. *vulgare*) | Spathulenol (20.7), *β*-caryophyllene (9.9) and caryophyllene oxide (5.7). | 0.03-0.06^f^ (dw ^d^) | ([Kula et al. 2007](#_ENREF_32)) |
| **Greece** | | | |
| Mountain Taygetos | Carvacrol (74.6), *p*-cymene (9.7), *γ* –terpinene (5.9). | 4.5^a^ (v/dw) | ([Karpouthis et al. 1998](#_ENREF_27)) |
| Iti mountain (subsp. *Hirtum*) | Thymol (45.2), carvacrol (33.1), *p*-cymene (7.4), *γ-* cymene (5.5). | 3.3^a^ (v/dw) | ([Adam et al. 1998](#_ENREF_1)) |
| **Serbia** | Carvacrol (61.3), thymol (13.9), *γ*–terpinene (3.1). | 1.5^a^ (dw^d^) | ([Bozin et al. 2006](#_ENREF_11)) |
| **Jordan** | *trans*-sabinene hydrate (27.2), terpineol-4 (19.4), *γ*-terpinene (7.8), *γ*-terpineol (6.6). | 0.23^l^ (dw^d^) | ([Al-Kalaldeh et al. 2010](#_ENREF_4)) |

^a^=dried/fresh aerial parts oil, ^b^=leaves and flowers oil, ^w^=whole plants oil, ^l^=leaves oil, ^f^=flowers oil, ^d^=Oil weight unit not reported, ^n^=plant parts not specified, N.R=not reported, tr=trace.

**
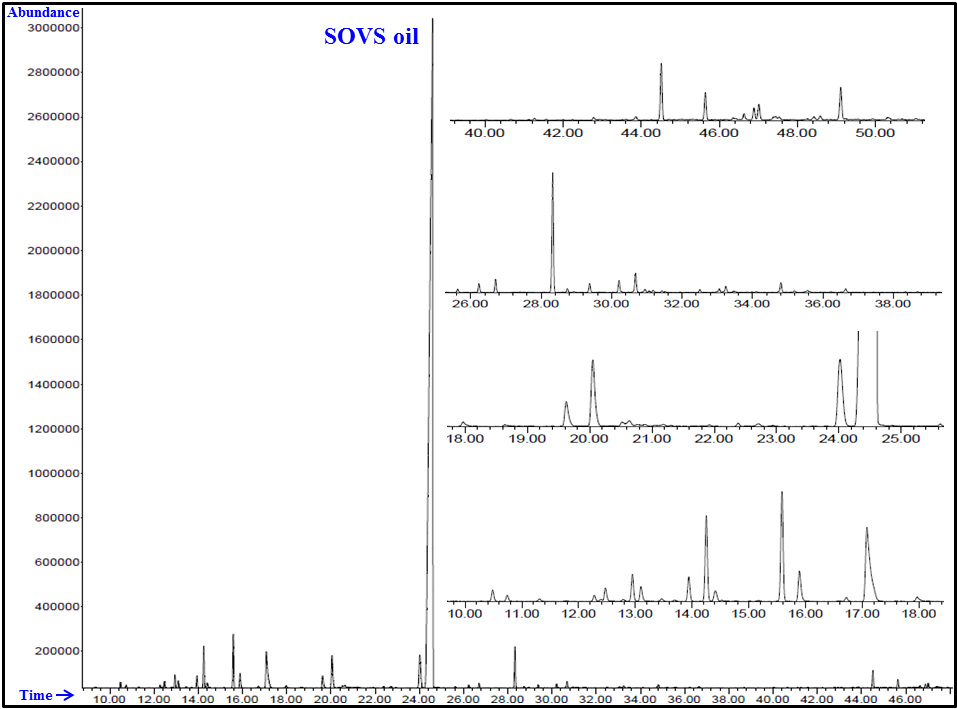
**

**Figure S1.** GC–FID chromatogram of essential oil from stems of Saudi *O. vulgare* L. obtained using HP-5MS column.

**
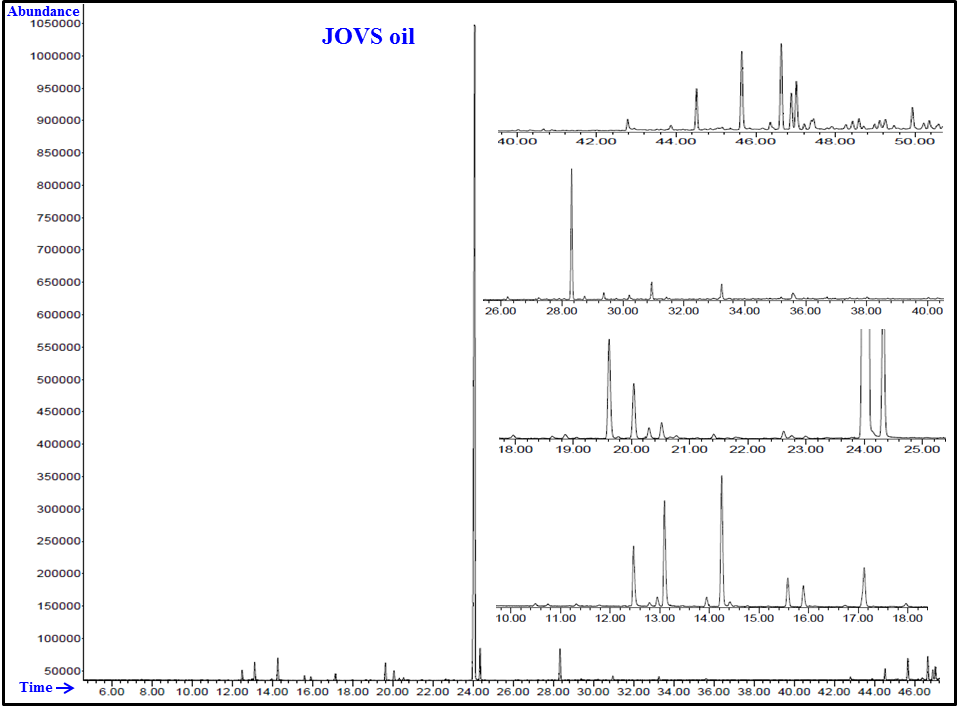
Figure S2.** GC–FID chromatogram of essential oil from stems of Jordanian *O. vulgare* L. obtained using HP-5MS column.


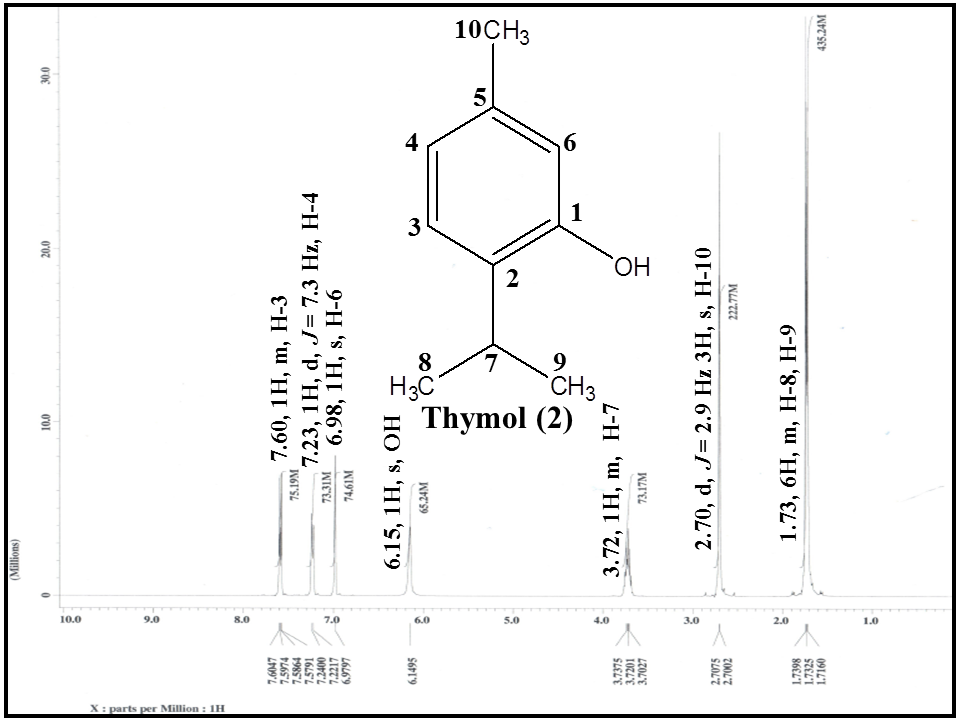


**Figure S3a.** ^1^H NMR spectrum of thymol (**2**) in CHCl_3_-*d*.


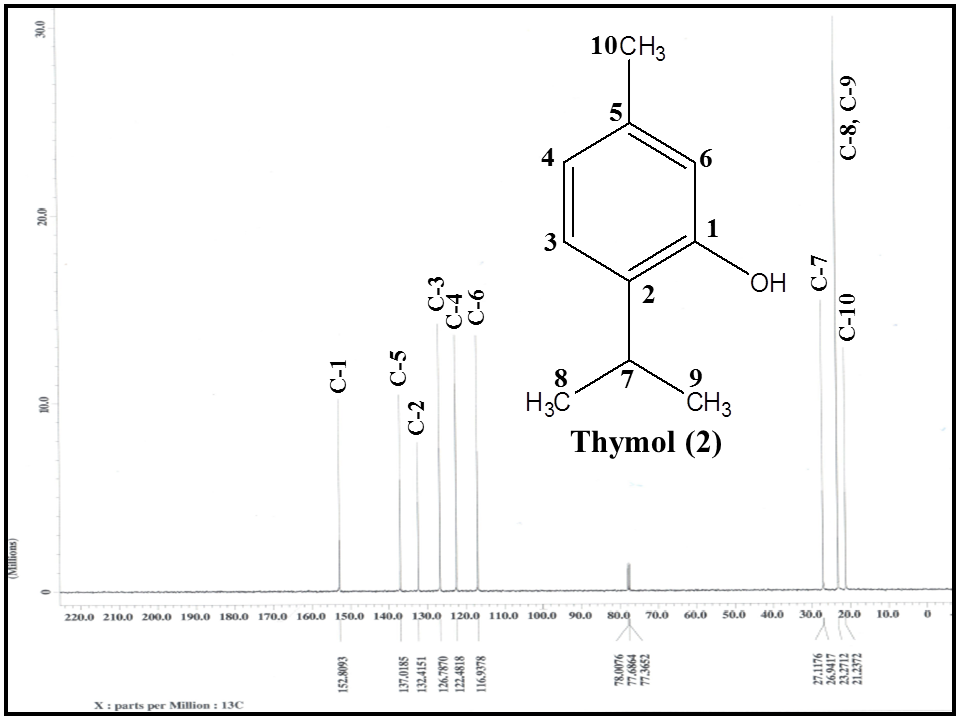


**Figure S3b.** ^13^C NMR spectrum of thymol **(2)** in CHCl_3_-*d*.

***S1.*** *Thymol*

White crystals. ^1^H-NMR (400 MHz, CHCl_3_-*d*): δ (ppm) = 1.73 (6H, m, H-8, H-9), 2.70 (3H, d, *J* = 2.9 Hz, H-10), 3.72 (1H, m, H-7), 6.15 (1H, s, OH), 6.98 (1H, s, H-6), 7.23 (1H, d, *J* = 7.3 Hz, H-4), 7.58 (1H, m, H-3); ^13^C-NMR (100 MHz, CHCl_3_-*d*): δ 21.2q (C-10), 23.3q (C-8, C-9), 27.1d (C-7),116.9d (C-6), 122.5d (C-4), 126.8d (C-3), 132.4s (C-2), 137.0s (C-5), 152.8s (C-1); EI-MS *m/z*: 150 ([M]^+^).

***S2.*** *Nuclear magnetic resonance (NMR) analysis*


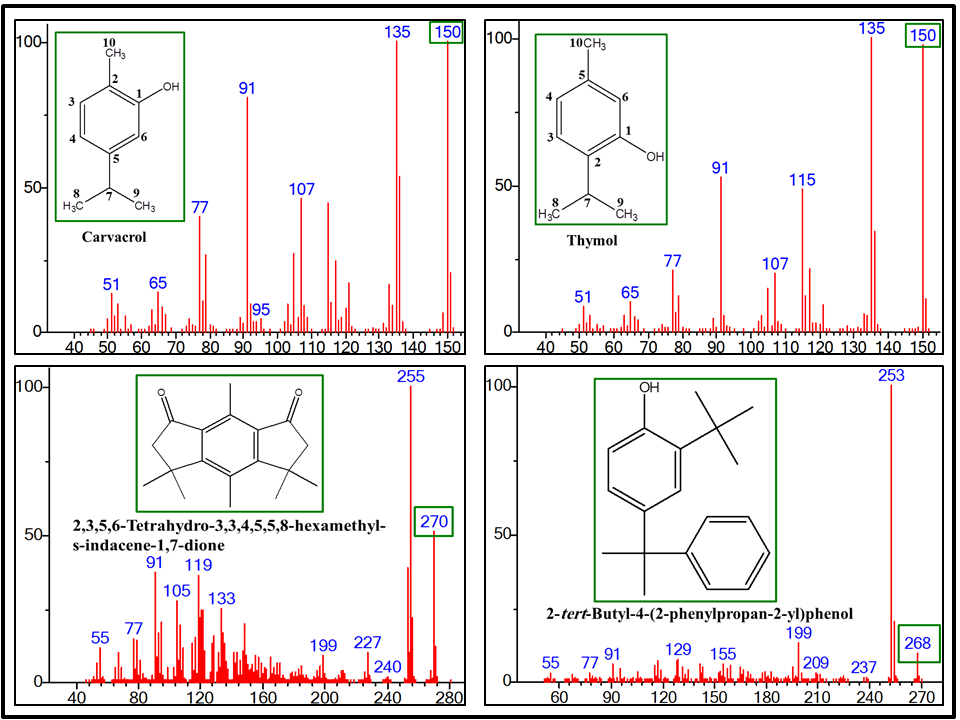
The ^1^H and ^13^C-NMR spectra of the pure compounds were obtained by using a JEOL ECP-400 spectrophotometer. The NMR samples were prepared in deuterated chloroform (CDCl_3_) and tetramethylsilane (TMS) was used as an internal standard. The chemical shifts and coupling constants (*J*) are expressed in *δ* (ppm) and Hz, respectively. Thin layer chromatography (TLC) was carried out on pre-coated silica gel 60 F_254_ (0.2 mm, Merck) plates and the compounds were detected under UV light.

**Figure S4.** EIMS fragmentation pattern of most dominant compounds in the essential oils of Saudi and Jordanian *O. vulgare* L.

**References**

Baser K, Özek T, Kürkçüoglu M, Tümen G (1994) The essential oil of Origanum vulgare subsp. hirtum of Turkish origin. J Essent Oil Res 6(1):31-36

Şahin F, Güllüce M, Daferera D, Sökmen A, Sökmen M, Polissiou M, Agar G, Özer H (2004) Biological activities of the essential oils and methanol extract of Origanum vulgare ssp. vulgare in the Eastern Anatolia region of Turkey. Food Control 15(7):549-557

Mechergui K, Jaouadi W, Coelho JP, Khouja ML (2016) Effect of harvest year on production, chemical composition and antioxidant activities of essential oil of oregano (Origanum vulgare subsp glandulosum (Desf.) Ietswaart) growing in North Africa. Ind Crops Prod 90:32-37

Gong H, Liu W, Lv G, Zhou X (2014) Analysis of essential oils of Origanum vulgare from six production areas of China and Pakistan. Rev Bras Farmacogn 24(1):25-32

Vazirian M, Mohammadi M, Farzaei M, Amin G, Amanzadeh Y (2015) Chemical composition and antioxidant activity of Origanum vulgare subsp. vulgare essential oil from Iran. Res J Pharmacogn 2(1):41-46

Moradi M, Hassani A, Sefidkon F, Maroofi H (2015) Chemical composition of leaves and flowers essential oil of Origanum vulgare ssp. gracile growing wild in Iran. J Essent Oil-Bear Plants 18(1):242-247

Mancini E, Camele I, Elshafie HS, De Martino L, Pellegrino C, Grulova D, De Feo V (2014) Chemical composition and biological activity of the essential oil of Origanum vulgare ssp. hirtum from different areas in the Southern Apennines (Italy). Chem Biodivers 11(4):639-651

Verma RS, Padalia RC, Chauhan A (2012) Compositional Analysis of the Leaf and Flower Essential Oils of Indian Oregano (Origanum vulgare L.). J Essent Oil-Bear Plants 15(4):651-656

Lukas B, Schmiderer C, Mitteregger U, Franz C, Novak J (2008) Essential oil compounds of Origanum vulgare L.(Lamiaceae) from Corsica. Molecular and phytochemical analyses of the genus Origanum L(Lamiaceae) 57(4):63

Nurzyńska-Wierdak R, Bogucka-Kocka A, Sowa I, Szymczak G (2012) The composition of essential oil from three ecotypes of Origanum vulgare L. ssp. vulgare cultivated in Poland. Farmacia 60(4):571-577

Ivask K, Orav A, Kailas T, Raal A, Arak E, Paaver U (2005) Composition of the essential oil from wild marjoram (Origanum vulgare L. ssp. vulgare) cultivated in Estonia. J Essent Oil Res 17(4):384-387

Kula J, Majda T, Stoyanova A, Georgiev E (2007) Chemical composition of Origanum vulgare L. essential oil from Bulgaria. J Essent Oil-Bear Plants 10(3):215-220

Karpouthis I, Pardali E, Feggou E, Kokkini S, Scouras ZG, Mavragani-Tsipidou P (1998) Insecticidal and genotoxic activities of oregano essential oils. J Agric Food Chem 46(4):1694-1694

Adam K, Sivropoulou A, Kokkini S, Lanaras T, Arsenakis M (1998) Antifungal activities of Origanum vulgare subsp. hirtum, Mentha spicata, Lavandula angustifolia, and Salvia fruticosa essential oils against human pathogenic fungi. J Agric Food Chem 46(5):1739-1745

Bozin B, Mimica-Dukic N, Simin N, Anackov G (2006) Characterization of the volatile composition of essential oils of some Lamiaceae spices and the antimicrobial and antioxidant activities of the entire oils. J Agric Food Chem 54(5):1822-1828

Al-Kalaldeh JZ, Abu-Dahab R, Afifi FU (2010) Volatile oil composition and antiproliferative activity of Laurus nobilis, Origanum syriacum, Origanum vulgare, and Salvia triloba against human breast adenocarcinoma cells. Nutr Res 30(4):271-278
